# Relationship between Frailty and Depression in a Population from North-Eastern Romania

**DOI:** 10.3390/ijerph19095731

**Published:** 2022-05-08

**Authors:** Alexandra Maștaleru, Irina Mihaela Abdulan, Ramona Ștefăniu, Nicoleta Lefter, Ioana Alexandra Sandu, Anca Iuliana Pîslaru, Maria Magdalena Leon-Constantin, Ioana Dana Alexa, Adina Carmen Ilie

**Affiliations:** 1 Department of Medical Specialties I, “Grigore T. Popa” University of Medicine and Pharmacy, 700115 Iași, Romania; alexandra.mastaleru@gmail.com; 2 Department of Medical Specialties II, “Grigore T. Popa” University of Medicine and Pharmacy, 700115 Iaşi, Romania; ramona.stefaniu@yahoo.ro (R.Ș.); lefter.nico@gmail.com (N.L.); ioana0sandu@gmail.com (I.A.S.); morosanu_anca@yahoo.com (A.I.P.); ioana.b.alexa@gmail.com (I.D.A.); adinacarmenilie@yahoo.com (A.C.I.)

**Keywords:** frailty, depression, geriatric assessment, quality of life

## Abstract

(1) Background: In the older population, depression often affects people with chronic medical illnesses, cognitive impairment, or disability. Frailty is another important issue affecting older adults, being difficult to clinically distinguish from frailty in advanced old age. Well-designed interventional studies and clinical strategies targeting both frailty and depression are rare or nonexistent. (2) Methods: We realized a retrospective study in which we included a total of 411 patients that were admitted to the Geriatric Clinic from “Dr. C. I. Parhon” Hospital from Iasi for a period of 13 months. The aim of our study was to investigate the relationship between depression and frailty in a geriatric population due to the fact that the quality of life is negatively influenced by both frailty and depression. (3) Results: The prevalence of the depressive symptoms screened by the GDS-15 was 66.7%, with women being more depressed than men. Furthermore, an obvious relationship between depression and the dependence degree in performing daily activities has been observed. In addition, the mean MMSE score decreased with an increasing degree of depression. (4) Conclusions: Our study demonstrates the association between frailty and depression, one of them being a risk factor in the development of the other. A poor acknowledgment of the problem and an underdiagnosis of these conditions are important public health concerns due to the high healthcare costs. Thus, an active primary prevention would be imperiously needed in order to diagnose frailty and depression at an early stage, increasing the quality of life of the elderly and also their successful aging.

## 1. Introduction

In the older population, depression often affects people with chronic disease, cognitive disorders, or disabilities. Consequently, it worsens the outcomes of many health problems and promotes malnutrition and isolation, beyond personal suffering and family disruption [1]. Several studies have found that a larger medical burden determines a greater chance of emotional impairment, thus making the elderly a particularly vulnerable group when it comes to depression [2].

Frailty is another important issue affecting older adults. Many symptoms such as poor activity, disability in performing daily activities, or low interest are common both in depression and in frailty. Further, frailty increases the risk of falls, disability, and hospitalization.

Recent interest in “overlapping syndromes” has led to the publication of some articles that demonstrate a positive correlation between the two [3,4]. This is not surprising given that frailty and depression have the “exhaustion” criteria. Examining this relationship is further complicated because the two syndromes “overlap” but are distinct [5,6]. There are a small number of studies that have included people over 65 years old who have been assessed for depression and frailty. The lack of unity in the diagnosis of the two syndromes and the lack of unique tools have led to extremely varied results. For example, Fried’s criteria, the most commonly used in the diagnosis of frailty, were not applied in all the mentioned studies, leading to different evaluations at various stages of frailty [3,4]. Furthermore, the definitions used were similar, but the exclusion criteria based on which the study groups were formed led to inconsistent results. One of the most common issues noticed was that when those with significant cognitive impairment were included, the rate of fragility and impairment of mood were higher [7].

The potential reversibility of frailty is an essential point, with a great impact on achieving successful aging. This concept is an ideal status in which patients will desire to have a high quality of life by minimizing their comorbidities and reducing the hospitalization for acute episodes, keeping their independence and also their social relationships, and promoting a healthy, active lifestyle. An early diagnosis by the general practitioner or geriatrician, together with comprehensive and correct management of frailty, would lead to successful aging, with a major positive impact on older people, their families and also society.

Furthermore, the available treatments for depression are as effective for old patients as for younger ones. However, early diagnosis of depression and immediate treatment remains an important goal. In conclusion, well-designed interventional studies and clinical strategies targeting both frailty and depression are rare or nonexistent.

The aim of our study was to investigate the relationship between depression and frailty in a geriatric population due to the fact that the quality of life is negatively influenced by both frailty and depression. Thus, their early diagnosis associated with active prevention would lead to successful aging.

## 2. Materials and Methods

**Figure 1:**
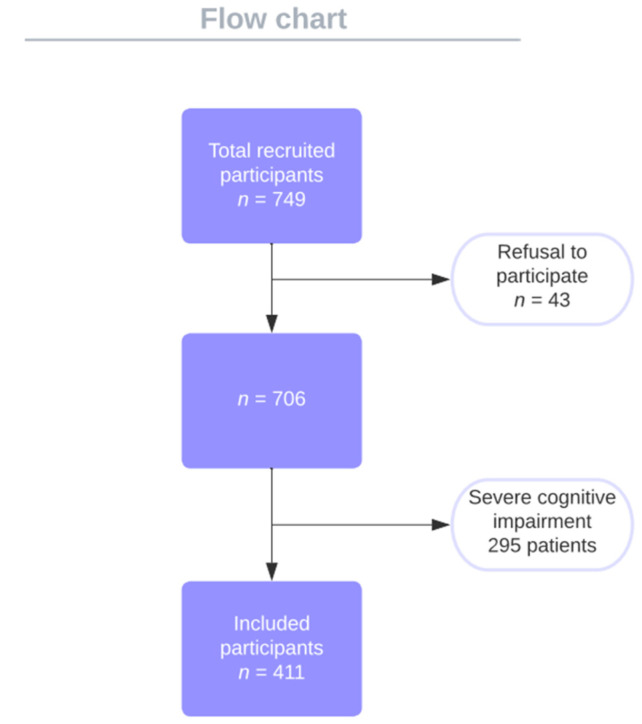
Flow chart of the studied group.

We realized a retrospective study in which we included a number of 411 individuals that were hospitalized in the Geriatric Clinic from the “Dr. C. I. Parhon” Hospital from Iasi between September 2013 and September 2014. Exclusion criteria were age under 65 years old, the refusal to participate, and severe cognitive impairment (Figure 1). Our study has been reviewed by the appropriate ethics committee from the University of Medicine and Pharmacy “Grigore T. Popa” Iasi, with MEN order number 183/16.04.2014, and has been completed in accordance with the ethical standards from the Declaration of Helsinki.

Frailty was assessed applying the original model, defined for the first time by Fried, which consists of five criteria: low walking speed, impaired grip strength, decreased physical activity, auto-reported exhaustion, and weight loss. If the patient had one or two criteria, they were considered as being pre-frail, while the presence of ≥3 criteria indicates the status of frailty. The geriatric depression scale (GDS) was used as an assessment tool for depression, where zero points indicated a normal state, and increasing scores were suggestive of aggravating symptoms.

Additional data were included: age, gender, rural/urban dweller, body mass index, compliance to treatment, number of drugs, and blood tests. The geriatric evaluation was included, as the doctors from our clinic have previously carried out these tests as part of a complete general examination.

The Katz Index of Independence in Activities of Daily Living (Katz ADL) is the most commonly used test to determine functional status and the status of independence. The functions tested are bathing, dressing, toileting, transferring, continence, and feeding. A score of 6 indicates full independency, 3–5 indicates moderate impairment, and 2 or less indicates the need for assistance.

For the instrumental assessment, we used The Lawton Instrumental Activities of Daily Living Scale (IADL). These skills included are more than those in the ADL, giving a good perspective of the patient’s status but also deterioration over time. There are 8 items: using the telephone, shopping, transportation, food preparation, housekeeping, laundering, handling medication and finances. The score ranges from 8 (independent) to 0 (dependent).

Depression was evaluated using the Geriatric depression scale (GDS), a short form that has 15 questions to assess our patient’s level of happiness, interest, and social communication. A score over 5 points indicates moderate depression, while a score over 10 indicates severe depression. The most common self-reported symptoms of the latter category were changes in appetite or weight, loss of energy, and feeling worthless.

The Mini Nutritional Assessment (MNA) was another part of the geriatric tests, a validated nutrition screening and assessment tool that can identify geriatric patients who are malnourished or at risk of malnutrition. We used the standard classification as follows: 24–30 p—normal status, 17–23.5 p—risk of malnutrition, below 17 p—malnutrition.

Cognitive status was the last aspect evaluated in the geriatric assessment. We used the Mini Mental State Examination, a 30 items test. Scores of 26 or higher are considered normal, 21–25 indicate mild impairment, 10 to 20 indicate moderate dementia. Patients with less than 9 points had a severe impairment and were excluded from the study.

Compliance with treatment was self-reported, and our patients were asked if they followed the recommended schemes. Their answers were noted as Yes or No. The number of drugs was also taken into account. Polypharmacy, a risk factor for depression and noncompliance, is defined as the daily use of at least 5 medications. More than 10 is considered excessive polypharmacy.

Blood tests were also included in the study. For hemoglobin, we used our laboratory cutoffs as follows: for females, the normal range was between 12 and 15.7 g/dL and for males between 13 and 17 g/dL. Anemia was considered for the patients who had values below this range and polyglobulia for values higher than the normal range. Regarding the white blood cells, leucopenia was considered in patients who had less than 4000/mm^3^, normal values were between 4000 and 10,000/mm^3^ and leukocytosis was considered over 10,000/mm^3^. For the erythrocyte sedimentation rate, a normal value was considered between 2 and 10/h, and a high value was in patients who had values more than 10/h. Additional data were included: age, gender, rural/urban dweller, body mass index, compliance to treatment, number of drugs, and blood tests. The geriatric evaluation included the cognitive state (Mini-Mental State Examination—MMSE), the degree of dependence/independence (Activities of Daily Living and Instrumental Activities of Daily Living), and nutritional status (Mini Nutritional Assessment—MNA). The doctors from our clinic have previously carried out these tests as part of a complete general examination.

**Figure 2:**
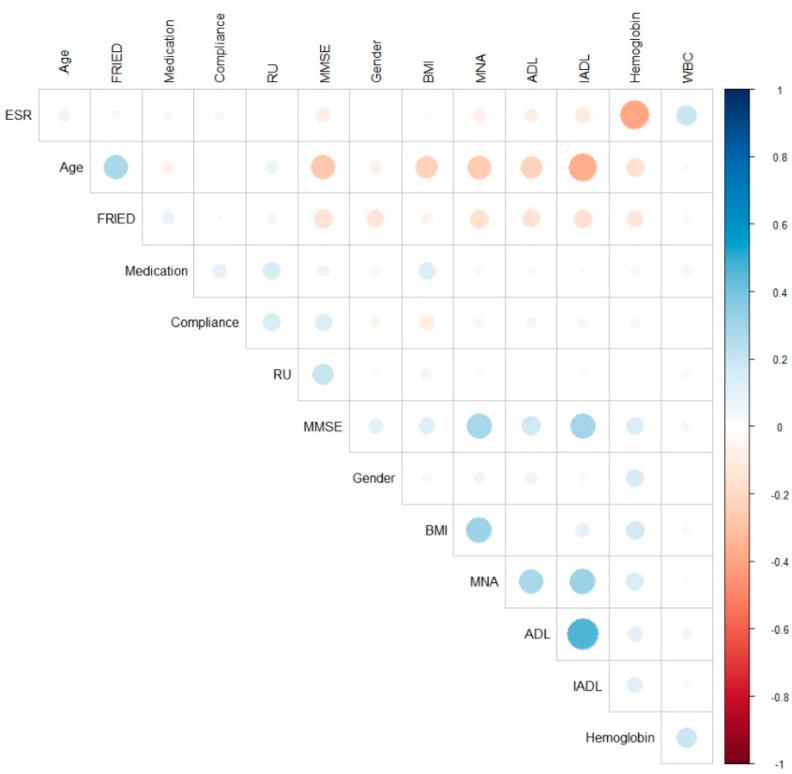
Correlations between geriatric assessment and anthropometric scores. ADL—activities of daily living, BMI—body mass index, ESR—erythrocytes sedimentation rate, GDS—geriatric depression scale, IADL—instrumental activities of daily living, MMSE—mini-mental state examination, MNA—mini nutritional assessment, RU—rural/urban area, WBC—white blood cells.

The statistical analysis was realized using SPSS Version 18.0. The profile of the patients included in the study has been described as means and standard deviation (SD), as medians and interquartile range (IQR), or as frequencies and percentages, in accordance with the type of variable (continuous, skewed continuous, and categorical). Multiple tests were performed: analysis of variance (ANOVA) for the descriptive indicators of the monitored variables, Chi-square test to correlate the frequency distributions, and Kruskal Wallis test to analyze 3 or more frequency distributions in the same study group. For Figure 2, we used the R program version 4.0.5. (R Foundation for Statistical Computing c/o Institute for Statistics and Mathematics Wirtschaftsuniversität, Wien, Austria).

## 3. Results

The general characteristics of the study group can be observed in Table 1.

Our patients had a mean age of 75.85 ± 6.35 years in a sample composed primarily of women (57.9%). Depression symptoms were observed in 66.7%. Women were more depressed than men (55.9% vs. 49.7% with moderate depression and 17.2% vs. 8.1% with severe symptoms). A diagnosis of major depression was assigned to 55 (13.4%) of the patients. The 134 subjects who were not clinically depressed had a mean score of 2.75 on the GDS, which was significantly lower than those with major depression, who had a mean of 12.07.

### 3.1. Distribution of Depression According to Geriatric Assessment

The correlations between geriatric assessment and depression can be observed in Table 2. Approximately one-quarter of the patients with severe depression were also malnourished.

In total, 74.2% of our subjects had various forms of malnutrition: 248 patients (60.3%) were at risk and 57 (13.9%) were malnourished. Cognitive impairment was found in 89.8%: 54.5% with mild and 35.3% with moderate cognitive disorder. A total of 2.2% were not able to perform ADL by themselves and 12.66% IADL (Table 2).

Most of the patients who needed assistance in performing daily activities had moderate depression: 88.9% (ADL), 67.3% (IADL). In total, 17.2% were diagnosed with moderate cognitive impairment and severe depression. Frailty was found mainly in those with moderate depression (58.1%).

### 3.2. Depression and Geriatric Assessment

There is an obvious relationship between depression and the dependence degree in performing daily activities: patients with a higher GDS score have low ADL and IADL values, a sign that they need help in performing basic daily and instrumental activities (Table 3).

The same situation is encountered in the case of the relationship between depression and cognitive impairment: the mean MMSE score decreases with an increasing degree of depression.

### 3.3. Correlations between Demographic Data and Geriatric Assessment

Linear correlations between geriatric assessment and the general patient’s characteristics are presented in Table 4 and in Figure 2. In Figure 2, we can observe negative correlations in red and positive correlations in blue. The sphere size and intensity are directly proportional to the correlation coefficients. Univariate correlations showed that gender, age, BMI, compliance, frailty, hemoglobin, and ESR significantly correlate with MMSE. All the geriatric scores were correlated with age, frailty, and hemoglobin level.

## 4. Discussion

Depression in older persons has many forms of manifestation, making it difficult to diagnose. It has a great impact on the quality of life and aggravates other comorbidities. Its presence increases the risk of cognitive impairment, disability and morbidity [8].

In our study, two-thirds of the patients were depressed, a higher percentage than in other similar studies. Community-based epidemiologic studies have reported different rates of depression, depending on the tools they used. Research that focused on symptoms of depression reported much higher prevalence rates than studies using diagnostic procedures.

Elevated scores in GDS were associated with frailty, varying from 10 to 20% [9,10]. Buigues et al. described in a study published in 2015 that approximately one-third of the patients diagnosed with frailty were also depressed and almost half of the frail older adults had mood disorders [4].

The association between depression and frailty was also found in previous studies [11,12]. This proves that the two conditions influence each other: fragile patients become depressed due to physical limitations, thus entering a vicious circle accentuated by other comorbidities. This association leads to poor outcomes such as disability and worse cognitive impairment [13].

Our research confirms a high prevalence of frailty, data that were also described in previously published research [14,15]. By definition, frailty occurs as the aging process evolves, resulting in a state of vulnerability and a decrease in the body’s reserves [16]. A meta-analysis from 2010 described that hearing loss, vision problems, stroke, cardiac disease, and chronic pulmonary diseases were conditions associated with depression and frailty [17].

Although frailty is a potentially reversible syndrome, there have been few interventional studies to date, and those that include depression are almost nonexistent. Several longitudinal studies have focused on the physical component of fragility, and there has been an improvement in physical activity after exercise. However, the criteria for frailty were different from Fried’s phenotype [5], making comparisons across results challenging.

Another noteworthy aspect was that overweight patients with a BMI between 25 and 29.99 had the lowest average depression score, compared to those in the extreme: underweight or those with obesity grade 3, the difference being statistically significant.

Whether we talk about obesity or underweight, malnutrition is one of the most important problems in older patients. It influences daily activities, muscular tonus but also metabolism or depression. Weight disorders increase the risk of mortality [18]. Although previous research has shown a clear link between weight and depression status, the common mechanism is unknown. In all age groups, including old individuals, weight changes can be a sign of depression. However, there are many people with constant weight over time diagnosed with emotional disorders.

The mechanisms that produce such an association seem to be different, depending on the age groups, if we consider that elderly patients are prone to suffer from multiple comorbidities, sarcopenia or dementia. Sad or disruptive events are also to be considered—retirement or death of a partner. The multitude of unknown elements requires targeted research to identify the factors that predispose the elderly to the association between depression and weight changes in order to implement early prevention and treatment measures.

Although we found no statistical significance, there is an increase in the average score of depression with age, so those in the age group 65–74 have a lower score compared to those in the group 85+. Depression is a syndrome that can affect an adult of any age. It can improve over time or become debilitating, so we face long-term depression. In addition, it can start in the last decades of life. This second variant is associated with an increased risk of developing cognitive impairment and dementia [19]. The incidence of depression also varies depending on the home environment, so the rate of depression among the self-employed at home is around 5–10%, while institutionalized patients have a higher rate (14–42%) [20].

The results presented in previous studies give us a valid justification for the high percentage of depression found in this research. In addition to therapy, the treatment of depression includes specialized medication. However, the geriatric patient is exposed to polypharmacy due to the large number of age-specific comorbidities, being itself a predictor of mood disorders [21]. Consequently, the early detection of depression, which can be treated initially non-pharmacologically, decreases the risk of polypharmacy.

In older patients, the pharmacological treatment for depression can be initiated if the non-pharmacological methods are not effective or sufficient, also due to polypharmacy and multiple drug interactions. At the same time, the correct treatment for depression brings about major benefits with regard to the evolution of other comorbidities and increases the compliance to the treatment and their quality of life. The results have immediate and particularly important practical implications, both in geriatric clinics and in general practitioner’s offices.

There is a statistically significant decrease in the average values of ADL and IADL with an increasing degree of depression. The results are consistent with the data presented in the literature. The relationship between physical dependence and depression is one of interdependence. The appearance of depression leads to the limitation of daily activities, which accentuates the depression. The aging process brings along many reasons to reduce physical activity or induce introspection and negative thoughts. In addition, some patients are more likely than others to decrease their activity and socializing moments when physical limitations or stressful episodes occur, especially people who have had an active lifestyle [22]. As we mentioned before, besides retirement, the loss of the partner is a major event because of the loss itself but also because of the tasks that must be performed by the remaining partner: housekeeping, cooking, and socializing. Family support may ease this transition, but for those who are helpless, the risk of self-neglect, isolation, and exacerbation of depression increases. In addition, isolation and lack of socialization bring with it self-critical thinking and low self-esteem.

The relationship between depression and cognitive impairment also showed statistical significance. Studies have shown that cognitive impairment worsens with age, being more pronounced in the age group over 85 years. However, the low addressability to the physician in this category of age and the association of the symptoms with the aging process leads to an underdiagnosis of dementia and delays the management of treatable diseases. In most cases, the mild cognitive impairment that accompanies a major depression event does not appear to proceed to dementia. In contrast, cognitive impairment is a stable disorder that only slightly improves when depressive symptoms subside [23,24].

We evaluated hemoglobin due to the fact that anemia can be a sign of malnutrition and also negatively influences the overall status and implicitly the quality of life by aggravating other comorbidities. A recent meta-analysis demonstrates that there is an important correlation between low hemoglobin and frailty, with the presence of anemia doubling the risk of developing this syndrome [25]. In our study, we observed a statistically significant correlation between anemia and cognitive status. Our results are in concordance with other recently published articles that observed that anemic patients have an increased risk of developing cognitive decline and dementia [26].

Regarding the inflammatory status, our results demonstrate a positive correlation between ESR, MMSE, and IADL. Previous studies have demonstrated that patients with high values of the ESR are prone to developing cognitive impairment and frailty; thus, our results are surprising. One explanation for this could be the fact that older people can have multiple causes of inflammation, such as rheumatological diseases, diabetes, atherosclerosis or chronic low-grade inflammation [27].

## 5. Conclusions

The presented data demonstrate the relationship between depression and frailty, one of them being a risk factor in the development of the other. Our results prove again the fact that interventional studies need to be performed on the geriatric population. Unlike other research, this study’s major advantage is the use of a comprehensive set of assessment tools for geriatric evaluation in older patients. 

We emphasize the importance of additional prospective studies in which specialized interventional measures (psychologist, nutritionist, physiotherapist) would slow the progression of the disease.

## 6. Limitations

The implications of this research are limited due to the subjectiveness of the tests. In addition, we have used the Fried auto-reported criteria without implementing the operational ones that will be used in future research. Moreover, the small number of studies focusing on frailty, depression or malnutrition and the lack of universal classifications or diagnostic tests still make the diagnosis of frailty impossible in the early stages.

## Figures and Tables

**Table 1:** General description of the study group.

	Depression					
	GDS	*p*	Normal	Moderate	Severe	*p*
Gender						
Female	6.71 ± 3.32	0.003	64 (26.9%)	133 (55.9%)	41 (17.2%)	0.001
Male	5.51 ± 3.28		73 (42.2%)	86 (49.7%)	55 (13.4%)	
Permanent residence						
Rural	6.28 ± 3.25	0.79	89 (34%)	142 (54.2%)	31 (11.8%)	0.473
Urban	6.48 ± 3.53		48 (32.2%)	77 (51.7%)	24 (16.1%)	
Age group						
65–74	6.03 ± 3.53	0.09	74 (40.4%)	85 (46.4%)	24 (13.1%)	0.153
75–84	6.48 ± 3.26		54 (28.9%)	107 (57.2%)	26 (13.9%)	0.578
85+	7.21 ± 2.77		9 (22%)	27 (65.9%)	5 (12.2%)	0.338
Body Mass Index (kg/m^2^)						
<18.5	8 ± 3.55	0.009	17.50 ± 0.86	17.20 ± 0.80	16.67 ± 1.14	0.54
18.5–24.99	6.72 ± 3.10		22.93 ± 1.42	22.43 ± 1.65	22.85 ± 1.67	0.22
25–29.99	5.63 ± 3.42		27.24 ± 1.52	27.17 ± 1.34	26.75 ± 1.18	0.52
30–34.99	6.29 ± 3.37		31.98 ± 1.39	32.49 ± 1.49	32.99 ± 1.36	0.13
35–39.99	7.33 ± 3.53		36.47 ± 1.51	36.95 ± 1.41	36.93 ± 1.22	0.77
>40	7.66 ± 3.46		41.72 ± 2.29	43.97 ± 3.42	40.12	0.43
Compliance with treatment						
No	6.53 ± 3.31	0.70	50 (34.2%)	76 (52.1%)	20 (13.7%)	0.933
Yes	6.26 ± 3.38		87 (32.8%)	143 (54%)	35 (13.2%)	
Number of medications						
<6	6.14 ± 3.31	0.29	78 (37.7%)	103 (49.8%)	26 (12.6%)	0.327
6–9	6.47 ± 3.21		48 (27.6%)	105 (60.3%)	21 (12.1%)	0.689
≥10	7.1 ± 4.36		11 (36.7%)	11 (36.7%)	8 (26.6%)	0.659
Hemoglobin						
Anemia	6.23 ± 3.30	0.25	38 (32.5%)	66 (56.4%)	13 (11.1%)	0.367
Normal	6.38 ± 3.41		98 (34.5%)	144 (50.7%)	42 (14.8%)	0.001
Polyglobulia	7.10 ± 2.37		1 (10%)	9 (90%)	0 (0%)	0.633
White Blood Cells						
Leucopenia	7.36 ± 3.38	0.80	2 (18.2%)	6 (54.5%)	3 (27.3%)	0.587
Normal	6.23 ± 3.30		108 (34.1%)	172 (54.3%)	37 (11.7%)	0.591
Leucocytosis	6.67 ± 3.53		27 (32.5%)	41 (49.4%)	14 (18.1%)	0.329
Erythrocite Sedimentation Rate						
Normal	6.23 ± 3.30	0.07	44 (36.4%)	63 (52.1%)	14 (11.6%)	0.781
High	6.40 ± 3.38		93 (31.1%)	156 (53.8%)	41 (14.1%)	0.102

**Table 2:** Geriatric assessment and depression.

	Depression			
	Total N = 411 (100%)	Normal N = 137 (33.33%)	Moderate N = 219 (53.28%)	Severe N = 55 (13.39%)
**ADL**				
Normal	278 (67.63%)	106 (38.1%)	148 (53.2%)	24 (8.6%)
Moderate	124 (30.17%)	31 (25%)	63 (50.8%)	30 (24.2%)
Severe	9 (2.2%)	0 (0%)	8 (88.9%)	1 (11.1%)
**IADL**				
Normal	159 (38.68%)	71 (44.7%)	71 (44.7%)	17 (10.6%)
Moderate	200 (48.66%)	59 (29.5%)	113 (56.5%)	28 (14%)
Severe	52 (12.66%)	7 (13.5%)	35 (67.3%)	10 (19.2%)
**MNA**				
Normal	106 (25.8%)	56 (52.8%)	44 (41.5%)	6 (5.7%)
Risk of malnutrition	248 (60.3%)	75 (30.2%)	138 (55.6%)	35 (14.1%)
Malnutrition	57 (13.9%)	6 (10.5%)	37 (64.9%)	14 (24.6%)
**MMSE**				
Normal	42 (10.2%)	20 (47.6%)	18 (42.9%)	4 (9.5%)
Mild	224 (54.5%)	87 (38.8%)	111 (49.6%)	26 (11.6%)
Moderate	145 (35.3%)	30 (20.7%)	90 (62.1%)	25 (17.2%)
**FRIED**				
Robust	44 (10.7%)	22 (50%)	18 (40.9%)	4 (9.1%)
Prefrail	66 (16.05%)	24 (36.4%)	26 (39.4%)	16 (24.2%)
Frail	301 (73.23%)	91 (30.2%)	175 (58.1%)	35 (11.6%)

ADL—activities of daily living, GDS—geriatric depression scale, IADL—instrumental activities of daily living, MMSE—mini-mental state examination, MNA—mini nutritional assessment.

**Table 3:** Depression and geriatric assessment.

	Depression			
	Normal	Moderate	Severe	*p*
**ADL**	5.72 ± 0.56	5.43 ± 1.02	5.16 ± 0.91	**0.0001**
Normal	6	6	6	-
Moderate	4.77 ± 0.49	4.60 ± 0.55	4.60 ± 0.55	0.31
Severe	0	3	3	-
**IADL**	6.86 ± 1.56	5.93 ± 2.04	5.83 ± 2.07	**<0.00001**
Normal	8	8	8	-
Moderate	6.03 ± 0.96	5.74 ± 1.04	5.78 ± 0.95	0.19
Severe	2.42 ± 1.13	2.31 ± 0.9	2.3 ± 0.67	0.92
**MNA**	21.01 ± 3.28	20.93 ± 4.13	20.3 ± 3.28	0.47
Normal	25.04 ± 1.69	25.4 ± 1.24	25.24 ± 1.68	0.51
Risk of malnutrition	20.54 ± 1.84	20.43 ± 2.39	20.01 ± 1.91	0.42
Malnutrition	14.73 ± 1.63	14.71 ± 2.17	14.9 ± 1.96	0.97
**MMSE**	25.83 ± 3.46	24.19 ± 3.56	24.01 ± 3.59	**0.000039**
Normal	30	30	30	-
Mild	26.7 ± 1.79	26.13 ± 1.72	26.23 ± 1.77	*0.07*
Moderate	20.56 ± 1.92	20.63 ± 1.77	20.76 ± 1.85	0.75
**FRIED**	2.60 ± 1.41	3.06 ± 1.26	2.78 ± 1.24	**0.005**
Robust	0	0	0	-
Prefrail	1.75 ± 0.44	1.61 ± 0.49	1.75 ± 0.44	0.52
Frail	3.46 ± 0.60	3.59 ± 0.62	3.57 ± 0.55	0.24

ADL—activities of daily living, GDS—geriatric depression scale, IADL—instrumental activities of daily living, MMSE—mini-mental state examination, MNA—mini nutritional assessment.

**Table 4:** Linear correlations between geriatric assessment and general characteristics.

	MMSE		MNA		ADL		IADL	
	r	*p*	r	*p*	r	*p*	r	*p*
Gender	0.109 *	**0.027**	0.062	0.208	0.067	0.177	0.032	0.521
RU	0.209 **	**0.000**	0.026	0.595	0.017	0.736	0.028	0.567
Age	0.272 **	**0.000**	0.255 **	**0.000**	0.224 **	**0.000**	0.369 **	**0.000**
BMI	0.124 *	**0.014**	0.321 **	**0.000**	0.024	0.633	0.108 *	**0.032**
Compliance	0.132 **	**0.007**	0.043	0.384	0.054	0.278	0.043	0.388
Medication	0.096	0.061	0.016	0.757	0.044	0.399	0.050	0.329
FRIED	0.156 **	**0.001**	0.160 **	**0.001**	0.150 **	**0.002**	0.165 **	**0.001**
Hemoglobin	0.128 **	**0.009**	0.146 **	**0.003**	0.106 *	**0.033**	0.118 *	**0.017**
WBC	0.047	0.339	0.027	0.589	0.053	0.287	0.037	0.452
ESR	0.103 *	**0.045**	0.092	0.071	0.092	0.072	0.115 *	**0.025**

Bold values indicate significant correlation (*p* < 0.05). ADL—activities of daily living, BMI—body mass index, ESR—erythrocytes sedimentation rate, GDS—geriatric depression scale, IADL—instrumental activities of daily Living, MMSE—mini-mental state examination, MNA—mini nutritional assessment, RU—rural/urban area, WBC—white blood cells. * *p* < 0.05; ** *p* < 0.01.

## Data Availability

Not applicable.
